# Elastic modulus of hyaluronic acid hydrogels by compression testing

**DOI:** 10.1007/s10856-025-06878-3

**Published:** 2025-07-14

**Authors:** Rachel Lee, Emily K. Hall, Bassam A. Aljohani, Jake McClements, Marloes Peeters, Mark Geoghegan

**Affiliations:** 1 School of Engineering, Newcastle University, Newcastle-Upon-Tyne, NE1 7RU UK; 2 Department of Physics and Astronomy, University of Sheffield, Sheffield, S3 7RH UK; 3 Chemical Engineering Department, Yanbu Industrial College, Yanbu Industrial City, Kingdom of Saudi Arabia; 4 Present Address: AkzoNobel, Stoneygate Lane, Gateshead, NE10 0JY UK; 5 Present Address: Unilever Research and Development Leeds, Coal Road, Leeds, LS14 2AR UK; 6 Present Address: Department of Chemical Engineering, University of Manchester, East Booth Street, Manchester, M13 9QS UK

## Abstract

Hyaluronic acid was crosslinked using 1-ethyl-3-(3-dimethylaminopropyl)carbodiimide and *N*-hydroxysuccinimide to form hydrogels with low elastic modulus. These hydrogels were swollen in water and the elastic modulus was obtained with a contact mechanics approach in ambient conditions using a low-load mechanical tester under compression. The modulus was measured during both the approach and retraction of the cylindrical probe into the gel and was found to be of the order of 30 kPa. The modulus was also measured from a stress-strain curve (47 kPa), in reasonable agreement with the contact mechanics approach. However, nanoindentation and rheology measurements reveal much smaller moduli, indicating that the technique used interrogates different length scales within the gel. This has profound implications for the applications of hydrogels used, for example, in tissue engineering. The values reported here are likely to be appropriate for applications where contact with the spinal cord is necessary. It is argued that a contact mechanics approach is appropriate for the characterization of hydrogels for applications designed for contact with tissue.

**Graphical Abstract:**

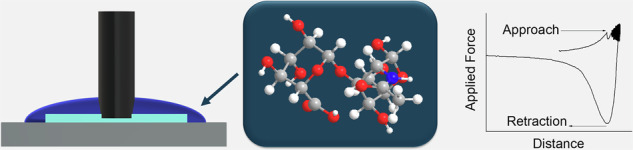

## Introduction

Hydrogels are three-dimensional networks of polymers which swell in water causing a large change in volume while maintaining network integrity in space through the means of crosslinks. These crosslinks can be physical or chemical and can be specifically designed to create scaffolds which can have softness, water content, and permeability similar to that of living tissue. Therefore, they are of great interest in biomaterial and medical studies [1, 2].

Depending on their function, the mechanical properties of hydrogels can be a critical parameter that needs to be controlled. For example, 3D scaffolds are frequently used in tissue engineering, where they provide a template for the regeneration of surrounding extracellular matrix and can be used as a host for cells or a device designed to manipulate a target area. Once placed into the body, such a device can carry out its intended function. Therefore, it is essential the scaffold has complementary properties to the target tissues. Knowledge of the mechanical properties of such a scaffold is essential to its design [3]. Hydrogels are often used in scaffolds because their physical properties can be adapted to the needs of the local physical environment. Crosslinks allow this by providing a means of control of the mechanical properties of the hydrogel [4].

A hydrogel to be used in tissue engineering requires an elastic modulus similar to the that of the tissues in the body. Compression testing measurements of the elastic modulus of a human cadaver spinal cord found an elastic modulus of ~40 kPa [5]. A survey of articles looking at the elastic modulus of animal cadaver spinal cords find moduli of ~9-25 kPa [6–8]. Therefore, an elastic modulus in the region of 5 to 50 kPa is a good target for a hydrogel to be suitable for applications involving contact with spinal cords.

Hyaluronic acid is ideal as a hydrogel scaffold due to its natural occurrence in the body, meaning it has inherent biocompatibility and can be crosslinked with a zero-length crosslinker to tailor its physical properties towards that of a specific tissue [9]. Furthermore, crosslinking density can be controlled using patterning technologies to give anisotropic behaviour, which is important where scaffolds of non-uniform shapes are required [10].

A micro-indenter is capable of measuring force and displacement simultaneously, and consequently provides a means of analysing the mechanical properties of a scaffold through a load-displacement relationship [11]. Deviations in the load-displacement relationship at the micro- or nanoscale have been defined as the normal contact problem [12, 13]. Hertz was the first to investigate the frictionless normal contact problem for deformable materials, noting the importance of the shape and position of the components in contact [13–15]. During compression, a material can be considered to extend in all directions. The region perturbed by an indenter as it moves from a boundary (surface) is known as the elastic half space, which is formally the semi-infinite volume below a surface of infinite lateral extent. The elastic modulus (E*) and Poisson ratio (*ν*) are required to describe the elastic half-space and are related by [16–18]

1 $$\frac{1}{{E}^{* }}=\,\frac{\left(1-{v}_{1}^{2}\right)}{{E}_{1}}+\frac{\left(1-{v}_{2}^{2}\right)}{{E}_{2}},$$

where the subscripts 1 and 2 refer to the medium under compression and the probe. E* is a reduced modulus accounting for these materials. A solution for solving the contact problem was provided by Sneddon, in which the load-displacement relationships for punch testing is defined by

2 $$F=a{h}^{m},$$

where *F* is the indenter load and *h* the elastic displacement. For a flat cylindrical indenter *m* = 1 [19].

Interest in identifying the elastic modulus of a medium began to increase during the 1970’s when a series of studies used microhardness indenter instruments to identify the load-displacement relationship [20, 21]. These were analysed in terms of the area of elastic contact *A*, the reduced elastic modulus E*, and stiffness, *S*, during unloading:

3 $$S=\,\frac{{dF}}{{dh}}=\,\frac{2}{\sqrt{{\rm{\pi }}}}{E}^{* }\sqrt{A}.$$

Equation (3) was originally proposed solely for use with conical indenters, although Bulychev later proved it is also valid for flat cylindrical indenters [13, 20, 21]. Equation (3) directly relates stiffness to the elastic modulus, which is important since the elastic modulus is often used as a proxy for the stiffness of the material in question.

A flat rigid indenter with the profile *f*(*r*), typically used for punch testing, can define the contact problem when it is combined with a contact quantity such as normal force *F*_N_, depth of indentation *d*, and the contact radius *a*. Flat indenters are typically used for punch testing. The profile *f*(*r*) is given by

4 $$f\left(r\right)=\left\{\begin{array}{c}0,\,r\le a\\ \infty ,r\,>\, a\end{array}\,\right..$$

When the contact radius is proportional to the radius of the indenter, which is the case for cylindrical indenters, a load-displacement relationship result, as described by [18], is given by

5 $$\frac{{dF}}{{dh}}=\,\frac{2}{\sqrt{{\rm{\pi }}}}{E}^{* }\sqrt{2{\rm{\pi }}{rh}+\,{{\rm{\pi }}r}^{2}},$$

which, when *h* is small, can be rearranged as,

6 $${F}_{{\rm{N}}}\left(d\right)=2{E}^{* }{da}.$$

In this contact mechanics approach where *r* = *a*, it is common practice to use *a* in Eq. (6). Traditionally when it comes to hydrogels, Eq. (6) is applied to the experimental results to extract the elastic modulus, since *h* ≪ *a* usually applies. The contact mechanics approach does not require any information about the size of the sample, assuming that the elastic half space assumption applies. It is nevertheless possible to obtain the elastic modulus of a material from the stress-strain curve, which may be compared to that calculated using contact mechanics.

Hydrogels are heterogeneous materials, which often gives them brittle character. If this heterogeneity is marked at the surface, it may lead to discrepancies in moduli measured from a stress-strain curve and those using contact mechanics. For applications that depend on material contact with tissue, contact mechanics is perhaps ideal because it reflects behaviour at the interface. With this consideration, it can be useful to compare the contact mechanics approach with the stress-strain behaviour in order to assess the effect, if any, of surface heterogeneity.

There have been a number of measurements of the mechanical properties of hyaluronic acid [22–29]. However, little attention has been given to a contact mechanics analysis of the mechanical properties, with the modulus coming from rheology [22, 24, 26], atomic force microscopy (AFM) nanoindentation [28], compression [23, 25, 27, 29], and tensile testing [29]. Of these, a contact mechanics analysis was only applied to the AFM experiment, which perturbs only the first few microns from the surface [30]. For the most part all experiments resulted in moduli of less than 100 kPa, although for some gels crosslinked using polymers significantly smaller moduli (<1 kPa) were recorded [22, 24].

In this work the elastic modulus of hyaluronic acid gels swollen to equilibrium in water and crosslinked using a zero length crosslinker is presented. The modulus is obtained using compression testing in a mechanical tester, which covers indentation depths of several hundred microns. This geometry is ideal for considering the mechanical properties of hydrogels that may need to be brought into contact with tissue. The contact mechanics method is assessed both for the approach of the indenter (probe) to the surface, which compresses the hydrogel, and its retraction from this compressed state. This allows an examination of any effect of hysteresis and also to mitigate experimental artifacts such as the probe penetrating surface water on the hydrogel. These results are compared with a typical stress-strain relationship and with experiments that interrogate different length scales: rheology and AFM nanoindentation. Both of these latter techniques offer different moduli. These differences provide unique insight into the complexity of hyaluronic acid, and provide some context on the challenges in reliably achieving some of the commercial applications [31].

## Methods

### Materials

Hyaluronic acid, 1-ethyl-3-(3-dimethylaminopropyl)carbodiimide (EDC), ethanol, and *N*-hydroxysuccinimide (NHS) were purchased from Merck and used as supplied.

### Synthesis of hyaluronic acid gels

A solution containing 1% (w/v) hyaluronic acid (0.1 g) in ethanol (2.5 mL), EDC (0.01 g), and NHS (0.04 g) was stirred for 20 minutes. Deionized water (7.5 mL) was then added dropwise while stirring and the mixture was left to stir for a further 40 min. HA is not soluble in ethanol, but it was not possible to disperse HA powder in pure water, so a mixture of ethanol is needed to create a film that is capable of effectively absorbing water [32]. A water rinse was used to wash out unreacted reagents. Following the reaction, the solution was left to dehydrate at room temperature for 48 h in an 80 mm diameter Petri dish. The dehydrated films were then cut into 1 cm^2^ pieces. The final swollen films were not uniform, but optical microscopy experiments indicated them to be between 0.5 and 1.5 mm in thickness. Pieces of HA gel were dried and swollen in water to measure the swelling ratio, which was determined as 10.5 ± 0.5 (the ratio of the mass of the swollen gel to that of the initial dry hydrogel).

### Measuring the stress-strain curve

A Stable Micro Systems TA.XTPlusC Texture Analyser (Surrey, UK) was set to measure the force applied to a sample under uniaxial compression in order to indent to a specified depth of 0.1 mm. The probe used was a cylindrical Delrin probe with a radius of 5 mm, an elastic modulus of 3.6 GPa, and a Poisson ratio of 0.35. The methodology of this test includes using a pre-test speed, a test speed, and a post-test speed of 0.5 mm/s respectively. Three samples synthesized under the same conditions were measured in all at ten different positions on each sample, so that the modulus is averaged over thirty values. The 5 mm probe width means that subsequent measurements were not all on surfaces that were untouched previously.

### Measuring the load-displacement profile for contact mechanics analysis

The TA.XTPlusC Texture Analyser was again used and was set to measure the depth of indentation at a specific applied force. Five samples synthesized under the same conditions were measured five times (each at a different point on the sample, with the caveat that each point does not preclude some overlap with another point) at intervals of 5 mN between 15 and 50 mN, which gives 25 data for each applied force. The same cylindrical Delrin probe was used as for the stress-strain measurements. The methodology for the experiment includes a pre-test speed of 30 mm/min, a test speed of 15 mm/min, a post-test speed of 30 mm/min, a hold time of 0.5 min, and a trigger force of 0 N. Here, it is assumed that the swollen gel is incompressible with a Poisson ratio, *ν*_1_ = 0.5.

### Rheological analysis

An Anton Paar modular compact rheometer (MCR 302e) was used to measure the storage and loss modulus of HA gels. A (20 mm diameter) parallel plate geometry was used during the test, where an equilibrium swollen HA gel of the same diameter was placed on the lower plate. Three samples synthesized under the same conditions were measured. First, the amplitude sweep test is performed by sweeping the strain (from 0.0001 to 1.0) to determine the linear viscoelastic region of the gel, which applied for shear strain less than 0.01. A frequency sweep across the range of 0.1 to 100 rad/s was therefore performed with a maximum strain of 0.01 to accurately measure the storage and loss modulus.

### Nanoindentation

Atomic Force Microscopy force-distance curves were acquired using a Bruker Nanowizard V (CA, USA) operating in force spectroscopy mode. All measurements utilized Bruker SAA-SPH-5UM cantilevers, which featured spherical tips with a nominal radius of 5 µm. The cantilevers were pre-calibrated with spring constants of 0.210 and 0.250 N/m, which are suitable for sensitive evaluation of synthetic hydrogels [33]. Measurements were conducted in deionized water with an approach/retract velocity of 2 µm/s and a force set point of 2.5 nN. Curves were collected at two different locations on the hydrogel in 11 × 11 square grids, each covering an area of 10 µm^2^, yielding 242 total force-distance curves. Data analysis was performed using the JPK Data Processing software, which included identifying the contact point between the AFM tip and hydrogel surface. The elastic modulus (*E*_1_) of the hydrogel was determined using the Hertizan model for spherical tips, defined by [34, 35]

7 $${F}_{{\rm{app}}}=\frac{4}{3}\frac{{E}_{1}}{1-{\upsilon }_{1}^{2}}{{R}^{1/2}d}^{3/2},$$

where *F*_app_ is the force applied by the tip, *R* is the radius of the spherical tip, and *d* is, as elsewhere, the indentation depth.

## Results

### Stress-strain curve

**Fig. 1:**
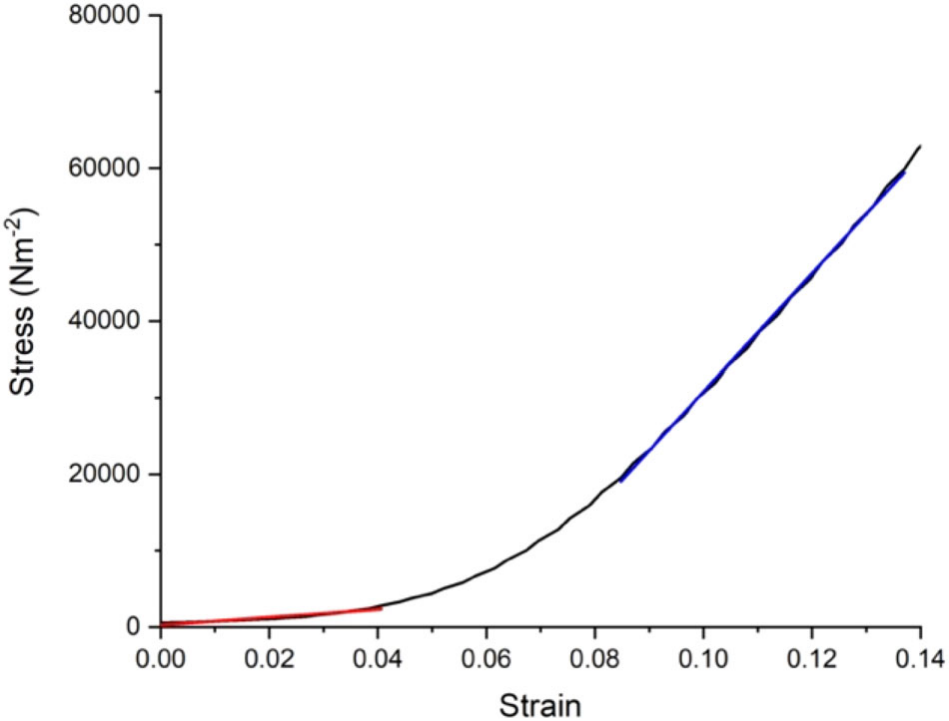
Stress-strain relationship during loading for a swollen gel of thickness 0.86 mm. Despite being in compression, the strain is shown as a positive number

The load-force relationship obtained by indentation testing can be plotted as a stress-strain graph (Fig. 1) of which the gradient provides the elastic modulus. For the hyaluronic gels used in this project the elastic modulus, obtained by repeat testing on each of three samples, is 46.8 ± 6.6 kPa. These results were taken from slope of the first linear region on the graph, where the probe is indenting into the sample to a specified depth of 0.1 mm.

The depth was chosen based on maintaining the elastic properties of the sample. The aim was not to penetrate the sample to the point of breakage, and so the test needed to end before plastic deformation. In some of the samples there are two distinct linear regions observed (Fig. 1): the first is related to the sample and the second appears to be where the data are affected by the probe interacting with the substrate underneath the sample. The elastic modulus of the second gradient is 0.7 ± 0.3 MPa which was attributed to the glass substrate. Certainly, a contact mechanics analysis of the bare glass substrate revealed a modulus of ~0.7 MPa. To obtain the stress-strain relationship the probe indents 0.1 mm into the gel and records the force it takes to reach this depth of indentation. Due to the thin film, the surface underneath the gel may contribute to the measurement. Therefore, the remainder of this article looks at examining the elastic modulus by specifying a force with the aim of minimizing the depth of indentation so that the surface underneath does not affect the result.

### Geometry for contact mechanics methods

**Fig. 2:**
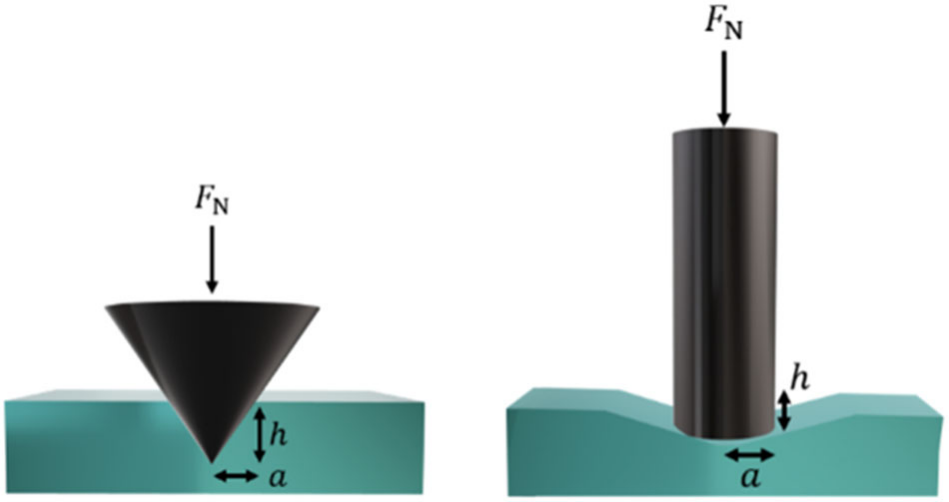
The indentation caused by probes with conical and cylindrical geometries

The gel is indented as the probe presses into the sample at a known force, which is modelled in terms of radius and the maximum depth of indentation (Eq. 6), as schematized in Fig. 2.

The model shown in Fig. 2, illustrates how the sample indents and diverges away from its normal state when it is compressed by a probe. In the example where the indenter used has a conical geometry the indent created by the probe is a complementary shape to the probe. The relationship between conical probe and indentation has been described in the work by Hertz and Boussinesq and has also been shown to be relevant to cylindrical and spherical probe geometries [14, 16]. In the case where the probe is a flat-ended cylinder, the sample is deformed by the probe at the flat end of the probe and the indent created is not such a complementary fit to the probe as it was for a conical indenter. Therefore, the model is not as accurate for flat indenters as it is for conical indenters. The model for both geometries is nevertheless usually described well by Eq. (6).

### Approach and retraction analysis

**Fig. 3:**
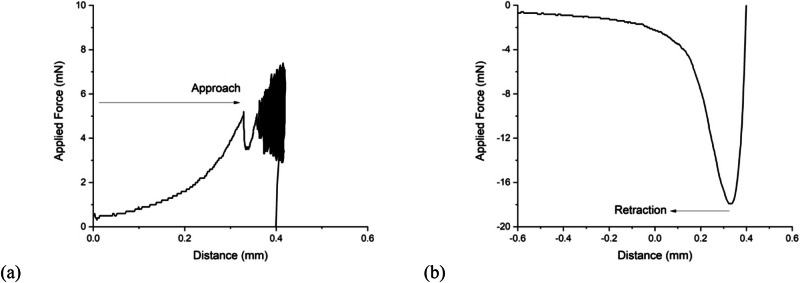
Detailing how the depth of indentation is seen in the raw load-displacement profile for a probe approaching and compressing the gel (a) and retracting away from the point of maximum compression (b)

A typical indentation experiment comprises three stages. First, the probe approaches the surface which it indents whilst maintaining a constant speed. In order to maintain constant speed, the force applied to the probe necessarily increases. At a specified force, the probe halts and the indentation depth is recorded (Fig. 3a), this being the difference between depth at which the target force was achieved and the point at which the probe first indented the sample. The modulus may be obtained from this (“approach”) method. At this point, the force is held constant to within experimental limits. Experimentally, oscillations are observed during this period, as can be observed at the largest depths in Fig. 3a. Finally, the probe retracts at constant speed and eventually leaves the hydrogel. Here the retraction distance is that travelled by the probe until it leaves the surface. The elastic modulus is also obtained during the retraction of the probe. The methodology is summarized below:

- **Approach**. The minimum point of indentation is the point of initial contact, and the gel is then compressed continually at an indentation speed of 30 mm/min until a target force is achieved; the maximum point is the furthest distance travelled by the probe. The distance between the maximum and minimum distance is the depth of indentation.
- **Retraction**. The maximum distance travelled by the probe is found at the point where the probe begins to retract from the gel. The minimum distance travelled by the probe is when the probe leaves the gel surface. The probe travels at a constant speed of 30 mm/min.

On approach, it is assumed that the first contact with the material is the point at which the first datum is recorded, also known as the trigger point. Therefore, the maximum distance the probe travels will provide the depth of indentation. The probe is incident on the sample, and first contacts a wetting layer on top of the hydrogel. The effect of the probe pressing through the surface of a small body of surface water is enough to trigger the device to begin to measure. This thin water layer presents a source of error.

On retraction, the indentation effects due to surface water can be more easily eliminated. The load-displacement profile of the curve describing the retraction has two parts (Fig. 3b). The first part of the curve is smooth and relatively linear, while the second has a shallower gradient. Here the steeper gradient is used, which eliminates errors due to the uncertainty as to the exact location of the surface of the hydrogel. Furthermore, a comparison between moduli obtained from both approach and retraction is a useful test of hysteresis in the data.

### Rheology of hyaluronic acid gels

**Fig. 4:**
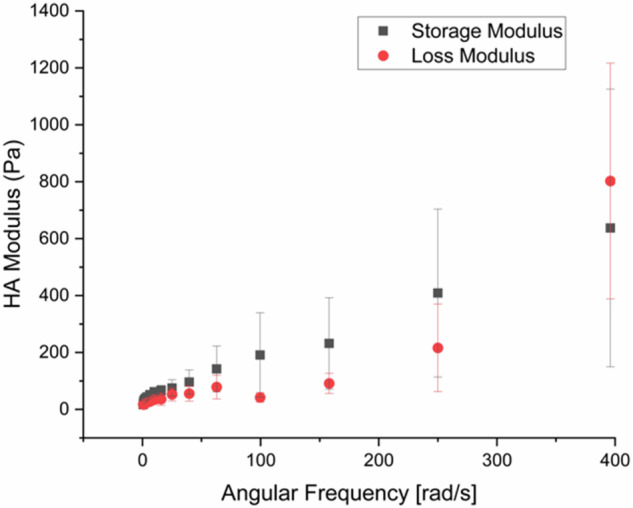
(a) The storage and loss modulus as a function of angular frequency obtained using a maximum strain of 0.01

Rheology experiments reveal a storage modulus starting at 17 ± 4 Pa, increasing with shear rate to 2.5 ± 1.5 kPa at the largest angular frequency of 628 rad s^–1^ (100 Hz). Figure 4 shows the results excluding this largest value, revealing the increase in the storage modulus with angular frequency. The loss modulus is finite at all frequencies, which indicates that pure elastic behaviour is not obtained in these experiments for these gels.

### Nanoindentation

**Fig. 5:**
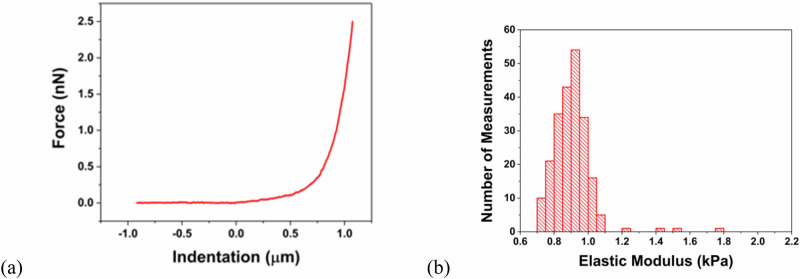
**a** Typical force curve showing the approach of the AFM tip to the hydrogel and subsequent indentation. **b** Histogram showing the distribution of elastic modulus values from the 247 measurements at different locations of the hydrogel

The AFM force spectroscopy measurements conducted at 242 locations on the hydrogel revealed a mean elastic modulus of 0.9 ± 0.1 kPa (Fig. 5). This value is calculated using *ν*_1_ = 0.5, assuming incompressibility, which is not necessarily the case for hydrogels fully immersed in water [36]. If *ν*_1_ = 0.33 were to be used, as was the case for poly(*N*, isopropylacrylamide) hydrogels [35], then the modulus increases to 1.1 ± 0.1 kPa. Such a small change does not affect the conclusions of the work. The incompressibility assumption is expected to remain valid for the compression testing experiments, where the swollen gel is not fully immersed in water.

## Discussion

**Fig. 6:**
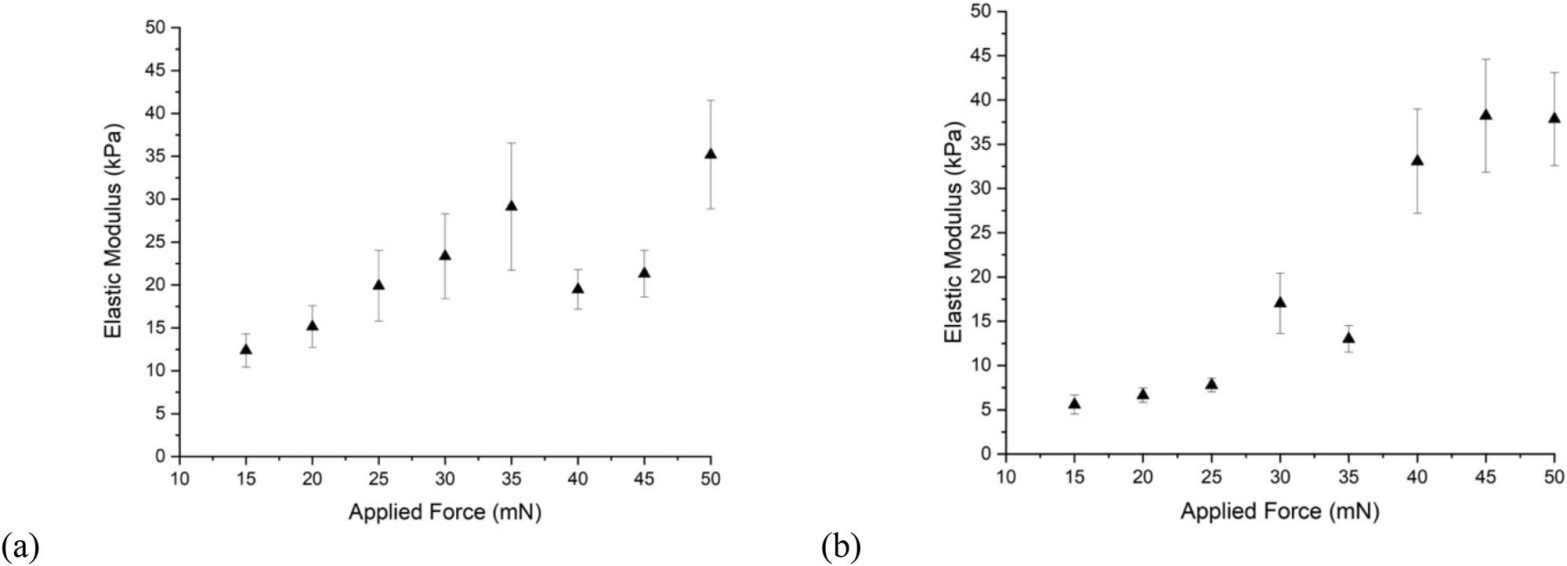
**a** The average elastic modulus, *E*_1_, at each interval of applied force calculated during the approach of the cylindrical probe. **b** The average elastic modulus at each interval of applied force calculated from data obtained during retraction of the probe. In both (**a**) and (**b**) data are the average moduli taken from 5 samples each tested in 5 different locations at each chosen interval of force

The load-displacement profiles over a series of known loads were obtained in both approach and retraction and from them elastic moduli were calculated. Equation (6) was used to calculate elastic moduli for each method. It was expected that the elastic modulus would be independent of load and experimentally this was observed for the approach method (Fig. 6a). Nevertheless, the data do suggest a small increase in modulus with increased final load.

The elastic modulus obtained from the approach data for an applied force of 15 mN is 12.4 ± 1.9 kPa and increases to a maximum of 35 ± 6 kPa at 50 mN.

For retraction, the mean elastic modulus is between 5.6 ± 1.1 and 38 ± 5 kPa (Fig. 6b) for applied forces of 15 and 50 mN respectively. The range of the elastic moduli is similar to that for data obtained during the approach part of the experiment. However, for these data, there is a stronger difference between smaller and larger applied loads.

Table 1 summarizes the elastic modulus for the approach and retraction contact mechanics methods at 15 mN and 50 mN. The elastic modulus at both loads is less than the 46.8 kPa result obtained from the stress-strain relationship of the hydrogel (also noted in Table 1), although there is agreement within error for the 50 mN load.

**Table 1:** The elastic moduli for each method at applied loads of 15 and 50 mN as well as the stress-strain result

	Method				
	Approach		Retraction		Stress-strain
Applied force (mN)	15	50	15	50	
Indentation (mm)					0.1
Elastic modulus (kPa)	12.4 ± 1.9	35.2 ± 6.3	5.6 ± 1.1	37.8 ± 5.3	46.8 ± 6.6

Rheology experiments reveal much lower moduli by orders of magnitude. Other rheometer measurements of HA have also resulted in very low storage moduli [22, 24], which are not inconsistent with the rheometer data presented here. The modulus obtained from nanoindentation is also smaller than that obtained from the contact mechanics approach presented here, but only by little over an order of magnitude. AFM nanoindentation measurements show that untreated hydrogels based on hyaluronic acid also exhibit an elastic modulus of ~1 kPa [28]. These hydrogels were synthesized with a light-sensitive molecular additive so that they could be stiffened by photo-exposure.

The difference in modulus for different testing methods is important. Nanoindentation probes the outermost surface of the gel, and it is plausible that here there are fewer complete crosslinks leaving the gel only partially formed. Contact mechanics is probing hundreds of microns and so presents a more robust surface test. However, rheology is capable of probing bulk properties. Although all of these techniques are interacting with the hydrogel in different ways, the differences in the values of the moduli indicate that care must be taken when designing a hydrogel for a given application.

## Conclusion

A standard contact mechanics approach, using force-displacement data, is compared with a stress-strain compression measurement using the same mechanical tester, rheology, and an atomic force microscopy nanoindentation experiment. The stress-strain relationship reveals similar values of modulus, of the order of 50 kPa, but the nanoindentation and rheology experiments reveal smaller moduli of ~1 kPa and 20 Pa, respectively. These large differences indicate that the scale at which the experiments interrogate the sample is important and must be considered for any application.

It is argued here, that for gels used as biological scaffolds, the hydrogel requires a conformal interaction with tissue and therefore a contact mechanics approach to measure the mechanical properties is appropriate. Here, using both the approach and retraction of the probe to identify the depth of indentation, any effects causing hysteresis can be identified, such as those arising from the probe interacting with surface liquid in the sample (used to maintain a swollen state) and the substrate.

The methods for testing the depth of indentation include measuring from the first datum to the maximum distance travelled when a set force triggered the experiment to stop. The second method (retraction) begins measuring the depth of indentation from the maximum distance travelled to the point at which the probe leaves the gel surface. Both of these methods have minor limitations which affect the obtained elastic modulus. However, the region over which the elastic modulus data fall is similar so there is no hysteresis in the data.

Although there is good agreement from the approach and retraction curves, the approach data are considered more reliable because they incorporate the distance from the probe’s first contact with the surface to the point at which a target force is met and therefore include the smallest strains. Although retracting the probe minimizes potential errors arising from determining the true surface of the gel, the data indicate that contributions from the substrate are more likely to be significant in retraction.

## Data Availability

The data that support the findings of this study are available from the corresponding author upon reasonable request.
